# The Use of Fissios App© as a Complement to a Face-to-Face Respiratory Physiotherapy Program versus an Attendance-Only Face-to-Face Physiotherapy Program in Patients Scheduled for Thoracic Surgical Procedures Reduces the Risk of Developing Postoperative Pulmonary Complications—A Quasi-Experimental Study

**DOI:** 10.3390/jcm12216774

**Published:** 2023-10-26

**Authors:** Carlos Alfredo Fraile Olivero, José Ramón Jarabo Sarceda, Elena Fernández Martín, Verónica Alen Villamayor, Joaquín Calatayud Gastardi, Ana María Gómez Martínez, Passio Santos, Pedro Daniel Arribas Manzanal, Carlos Cerdán Santacruz, Florentino Hernando Trancho

**Affiliations:** 1 Thoracic Surgery Department, Hospital Clínico San Carlos, 28040 Madrid, Spain; joseramon.jarabo@salud.madrid.org (J.R.J.S.); jcgastardi@hotmail.com (J.C.G.); anamagom@ucm.es (A.M.G.M.); florentino.hernando@salud.madrid.org (F.H.T.); 2 Physical Medicine and Rehabilitation Department, Hospital Clínico San Carlos, 28040 Madrid, Spain; 3 Department of Coloproctology, Hospital Universitario de la Princesa, 28006 Madrid, Spain

**Keywords:** perioperative care, preoperative exercise, breathing exercises, postoperative complications, length of stay, thoracic surgical procedures, mobile applications

## Abstract

Postoperative pulmonary complications (PPCs) increase the hospital length of stay (LOS) and the cost of healthcare associated with surgical procedures. Strategies to reduce PPCs begin before surgery and continue in the postoperative period. Fissios App© is a smartphone application that contains perioperative medical advice and a structured respiratory physiotherapy program. The objective was to implement the use of this app in a group of patients scheduled for a thoracic surgical procedure and determine its efficacy in reducing PPCs. This was a quasi-experimental study in which all patients attended a face-to-face respiratory physiotherapy program, and the intervention group used Fissios App© as a complement. We prospectively recorded the postoperative evolution of both groups, analyzed the categorical differences and quantitative variables, and created a binary logistic regression model. We recruited 393 patients (131 intervention and 262 control). The intervention group had a lower incidence of PPCs (12.2% versus 24% in the control group, *p* = 0.006), a shorter LOS (a median of 3 days (IQR = 2–5) versus 4 days (IQR = 3–6, *p* = 0.001) in the control group), and a reduction in the risk of developing PPCs by 63.5% (OR: 0.365, 95% CI: 0.17–0.78). The use of Fissios App© improved the clinical outcomes after surgery and reduced the probability of developing PPCs.

## 1. Introduction

Despite advances in perioperative care, postoperative pulmonary complications (PPCs) after thoracic surgical procedures remain prevalent. They are related to the increasing hospital length of stay (LOS), the cost of healthcare associated with surgical procedures, and mortality [1,2]. Furthermore, surgically treated nonsmall cell lung cancer (NSCLC) patients who overcome PPCs have worse overall and disease-free survival [3]. The implementation of multimodal evidence-based perioperative strategies can enhance postoperative recovery and is associated with improved clinical outcomes after surgery [4].

Preoperative exercise-based training improves pulmonary function before surgery and reduces hospital LOS and PPCs after surgery for NSCLC [5,6]. However, there is heterogeneity in the interventions prescribed (modality of exercise, mode of delivery, frequency, duration), and the majority of these programs are facility-based interventions [6]; attending these programs might be one barrier to reaching the adherence needed [7]. Further studies with defined exercise programs, larger samples, and higher methodological quality are required to clarify the potential benefits of preoperative exercise training before surgery [8].

Fissios App© (Tribalyte Technologies, Madrid, Spain) is a smartphone application created by a multidisciplinary working group composed of thoracic surgeons, physiotherapists, and a physiatrist. It contains a structured respiratory physiotherapy program with 10 exercises (including aerobic activity and respiratory muscle training) and 40 multidisciplinary perioperative medical advice. This study aimed to implement the use of Fissios App© as a complement to a face-to-face respiratory physiotherapy program in a group of patients scheduled for a thoracic surgery procedure and determine its efficacy in improving postoperative outcomes in terms of reducing PPCs and shortening LOS.

## 2. Materials and Methods

This is a prospective and quasi-experimental study with an intervention and a control group. Consecutive sampling was selected; during the study period, all patients who attended the thoracic surgery outpatient department and were scheduled for surgery could be part of the sample. It was conducted in a tertiary university hospital in Madrid, Spain, between June 2017 and December 2018. We adhered to the “TREND” (Transparent Reporting of Evaluation with Nonrandomized Designs) Statement. The realization of this study has the approval of the local research ethics committee (code: 16/117-E).

Intervention: All patients who attended outpatient clinics received information about the study, verbally and in writing with a “patient information” document (Supplementary Material File S1). Those who decided to participate received more detailed instructions to download and install Fissios App© (Supplementary Material File S2). The included patients followed the same protocol established in our department: After an evaluation by a physiatrist, who prescribed the exercises, they attended one-hour face-to-face respiratory physiotherapy sessions taught by physiotherapists at the hospital five times per week before the planned surgery and continued with the same frequency during the postoperative period until discharge. The physiotherapy sessions included breathing exercises, usage of incentive spirometry, and coughing exercises. The control group only attended face-to-face respiratory physiotherapy sessions; the intervention group attended these sessions, and they were encouraged to use the app before surgery, accomplish the medical advice, and perform the respiratory physiotherapy program. Fissios App© contains a structured respiratory physiotherapy program with 10 exercises (including aerobic activity and respiratory muscle training) and 40 multidisciplinary perioperative medical advice (Table 1). Adherence to this tool was monitored by the physiotherapist trained to check the completion of the exercises on patients’ devices.

All surgical procedures were performed under general anesthesia and selective orotracheal intubation with a double lumen tube, including conventional (posterolateral thoracotomy) and minimally invasive (2–3 ports VATS) approaches. The operations were undertaken by the same team of certified thoracic surgeons with homogeneous criteria regarding the approach, type of resection, and postoperative care protocols.

Criteria for eligibility: The patients included were all those aged over 18, with a pathology requiring a programmable thoracic surgical intervention for diagnostic or therapeutic purposes, candidates for respiratory physiotherapy, and the will to participate in the study. The exclusion criteria were defined as (1) emergency surgery, (2) patients not candidates for surgery, (3) patients physically or mentally not suitable to perform respiratory physiotherapy exercises, and (4) refusal to participate in the study. Patients who met the inclusion criteria and agreed to participate signed the informed consent.

Allocation of patients to groups: The allocation was not randomized. The intervention group consisted of all those patients who, meeting the inclusion criteria and none of the exclusion ones, had an intelligent mobile device compatible with Fissios App© and were able to perform the exercises without supervision. Those patients who did not have an intelligent mobile device compatible with Fissios App©, were not suitable to perform respiratory physiotherapy exercises without supervision, or were not able to install or use the app were automatically allocated to the control group. All surgeons in the service were trained to invite all patients to participate and allocate them to the appropriate group. To confirm that the patient had clear participation in the study and could use the app without problems, the possibility of attending a new medical visit in the clinics was allowed.

Sample size calculation: Based on the percentage of PPCs developed in 2016 at our Thoracic Surgery Department (22.8%) and the expected reduction in the intervention group (50%), the sample size was calculated in each group to make a comparison of two proportions. To achieve a power of 80% and detect differences in the contrast of the hypothesis using a two-sided χ^2^ test for 2 independent samples, taking into account that the level of significance is 5%, it was necessary to include 255 units in the control group and 131 units in the experimental group.

Outcomes measure: The primary clinical endpoint was postoperative pulmonary complications. They were recorded as the onset of at least one adverse clinical event, including air leak >5 days, bronchoscopy for atelectasis, pneumonia, acute respiratory distress syndrome, initial ventilation support >48 h, reintubation, tracheostomy, pleural effusion, pneumothorax, empyema, and pulmonary embolus. We recorded all complications that occurred within 30 days or during a longer period if the patient was still in the hospital and were defined according to the STS-ESTS joint standardization of variable definitions [9]. The secondary clinical endpoints were hospital LOS, 30-day mortality, the time of use of Fissios App© before surgery, and the probability of developing PPCs. All variables were collected prospectively through medical records during hospitalization and completed on the 30th day of postoperative consultation at the thoracic surgery outpatient clinics.

Other variables of interest: Demographic data from patients, including sex and age, were recorded. Smoking status and the American Society of Anesthesiologists (ASA) grade were collected too. Surgical procedure data such as lung parenchyma resection and surgical approach were included in the study variables.

Statistical analysis: The normal distribution of variables was first assessed by the Kolmogorov–Smirnov normality test. Qualitative variables are presented as frequency distribution with a 95% confidence interval (95% CI). Quantitative variables with normal distribution are presented as mean ± standard deviation (SD), and non-normally distributed data as median and interquartile range (IQR). Categorical variables were tested by the chi-square test or the Fisher exact test. We analyzed the behavior of quantitative variables for each of the independent variables categorized by the Mann–Whitney U test and adjusted a logistic regression model to identify and evaluate the relationship between the explanatory variables and the occurrence of the event. The probabilities of the presence of the events of interest were estimated based on the explanatory variables that were independently associated with the event according to the multivariable analysis. In all hypothesis tests, the null hypothesis was rejected with a type I error or α error of less than 0.05. Statistical tests were performed on the statistical software SPSS 20.0 (IBM Corp. Released 2011. IBM SPSS Statistics for Windows, Version 20.0. Armonk, NY, USA: IBM Corp.).

## 3. Results

**Figure 1:**
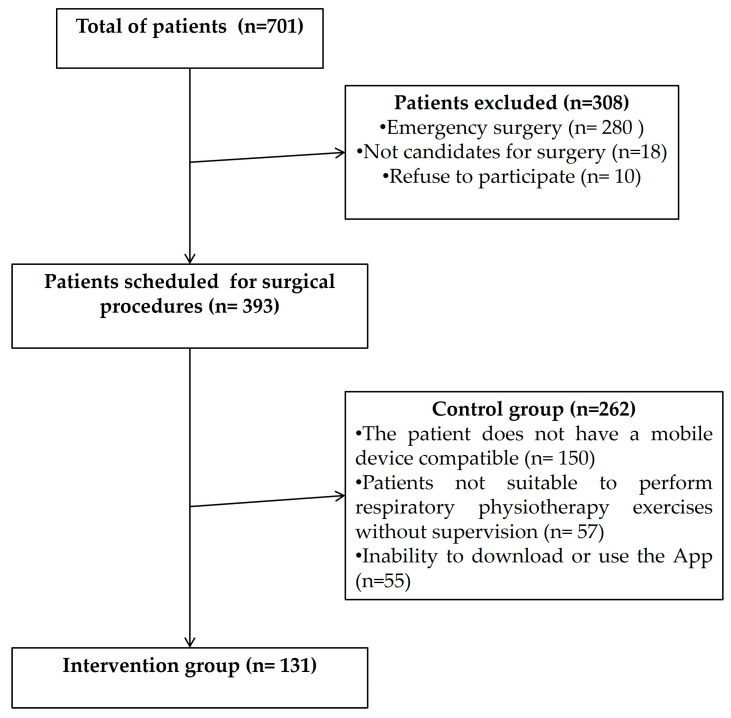
Flowchart of patients through the study.

This study recruited a total of 393 patients scheduled for surgery between June 2017 and December 2018, with a distribution of 131 patients in the intervention group and 262 in the control group. Patients scrutinized for eligibility and the definitive study sample are shown in Figure 1.

The intervention group had a lower median age (62 years) and a higher proportion of patients with lung parenchyma resection surgery (87.8%) compared with the control group. No differences were found in terms of smoking status, American Society of Anesthesiologists (ASA) grade, or surgical approach (Table 2).

**Figure 2:**
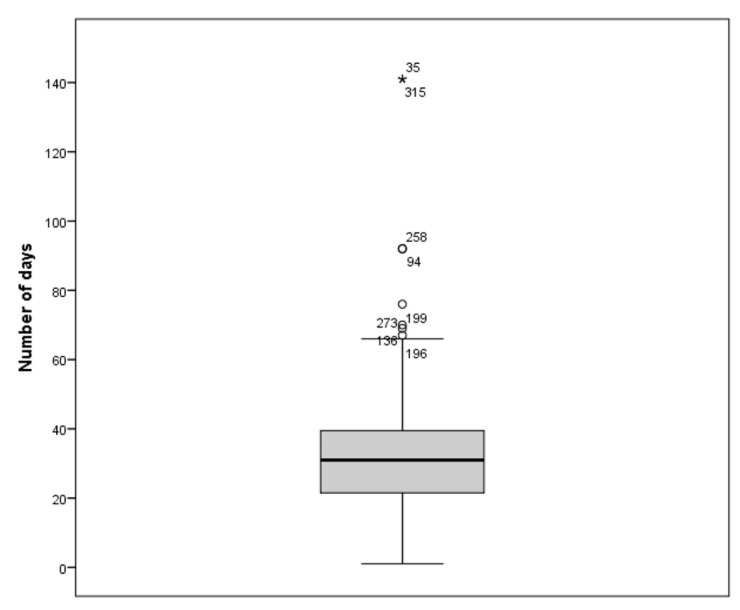
Number of days that patients used Fissios App© before surgery. * represents 2 outliers patients who used Fissios App© more days than the rest of patients.

The patients in the intervention group used Fissios App© more than 4 weeks before surgery, with a median of 31 days (IQR = 21–40) (Figure 2). During the study, all patients who interacted with Fissios App© did not report any adverse effects or technical problems associated with its use.

The incidence of PPCs was lower in the intervention group (12.2% versus 24% in the control group, *p* = 0.006), the most frequent event recorded was pneumonia, and the incidence of air leak was higher in the control group (8.4% versus 3.1% in the intervention group, *p* = 0.004). The intervention group had a shorter postoperative LOS, with a median of 3 days (IQR = 2–5) versus 4 days (IQR = 3–6, *p* = 0.001) for the control group. The two groups did not differ regarding postoperative mortality (Table 3).

A binary logistic regression model, adjusted for age and sex, was created to define the probability of developing PPCs. This model was significant, and it correctly classified 79.9% of the cases. The independent variables analyzed were sex, age, surgical approach, lung resection surgery, and the use of Fissios© for more than 4 weeks. In the adjusted analysis, the variables associated significantly with the probability of developing PPCs were male sex (OR: 2.3; 95% CI: 1.3–4.2), conventional surgical approach (OR: 2.5; 95% CI: 1.4–4.49), and lung parenchyma resection (OR: 4.2; 95% CI: 1.76–10.21). In addition, taking into account sex and age, the patients who used Fissios App© for more than 4 weeks reduced the risk of developing PPCs by 64% compared with those who did not use it (OR: 0.365; 95% CI: 0.17–0.78) (Table 4).

## 4. Discussion

The use of a remote and home-based respiratory physiotherapy program with Fissios App©, as a complement to a face-to-face program, reduced the incidence of PPCs (air leak, pneumonia, and atelectasis) and the probability of developing them by 64%, and shortened the hospital stay by 1 day.

Perioperative exercise training has been demonstrated to reduce postoperative LOS and the incidence of PPCs after surgery for NSCLC [5,9]. However, these results must be interpreted with caution due to the heterogeneity of the published studies, the lack of consistency in the exercise regimens, and the small sample sizes of the studies [10]. It is recommended to launch randomized multicenter trials with defined physical exercise programs and a more homogeneous sample to strengthen the existing scientific evidence [8,11]. In addition to physical exercise, other strategies are effective in enhancing recovery and improving surgery outcomes [4,12], so there is an interest in the development and implementation of multimodal perioperative programs [13,14]. Our study was carried out only in thoracic surgery patients and in a single center with standardized postoperative care protocols to obtain a significant and homogeneous sample. Fissios App© is a multidisciplinary tool that contains a structured respiratory physiotherapy program and medical advice with other strategies (stop smoking, balanced diet, oral care) that have been shown to improve outcomes after surgery. Together, it focuses on perioperative patient education to help them face surgery and enhance their recovery.

In this study, we reported a lower incidence of postoperative complications in the intervention group (24% vs. 12.2%). These results are similar to other studies that used a multidisciplinary approach, reducing the incidence of postoperative pneumonia and the need for reintubation and mechanical ventilation [15]. The strength of these programs lies in the sum of the benefits and the synergistic effect they generate. If they are studied individually, the results could be contradictory [16]. We were also able to observe a reduction in hospital LOS; the postoperative LOS was 1 day shorter in the intervention group. Our results fit with other studies, in which respiratory physiotherapy programs are associated with a decrease in hospital LOS [5,6,17]. Finally, we are the first group to introduce the use of a smartphone app as an independent factor associated with a decreased risk of PPCs in a multivariate analysis adjusted for age and sex. There is no possibility to compare our results with other similar studies because we are the first to introduce the performance of exercises of respiratory physiotherapy with the use of an app in a group of patients undergoing thoracic surgical procedures.

Age is a predisposing factor for the development of PPCs after lung resection surgery, and it has been recognized as an independent prognostic factor in relation to air leak [18]. Older patients are more likely to develop complications due to associated comorbidities or the ability of the body to respond to a stressful situation, such as surgery. In our study, the univariate analysis showed that the intervention group had a lower median age, and this could be associated with a lower complication rate. However, a logistic regression model was created, and in this multivariate analysis, age was not associated with an increased risk of developing PPCs. The results of our study support other publications in which other associated risk factors exist, such as ASA grade, surgical technique, or performance status of the patients [19]. Age alone can influence the development of PPCs but is not decisive and, thus, it is not a criterion to exclude a patient from surgery. We must pay attention to the other comorbidities of the patient, nutritional status, and even mental state because when several factors are combined, they can have a synergistic effect and favor the appearance of complications.

In this study, 150 patients did not have a compatible mobile device, and 55 patients had a compatible device but could not download or use the app. This was a significant number of patients and required an analysis of the causes of this phenomenon, such as age, socioeconomic level, or educational level. Fortunately, our team analyzed, in a prior study, the sociodemographic characteristics of patients in the intervention group, and we demonstrated that neither age nor the educational level of patients limited the implementation and use of the app in our department [20]. However, in recent years, with the rise of new technologies and mobile apps, most devices are compatible with simple apps, and patients are often more familiar with the use of these technological tools.

Once surgery is indicated, preoperative respiratory rehabilitation should be started as soon as possible to ensure compliance with therapy without delaying surgery. Although some studies have shown beneficial results with short 1-week exercise regimens [21], most studies reported at least 4 weeks [5]. In our study, most patients used the app with a median of 31 days before surgery; the achievement of this objective may be attributed to the fact that there was no delay to the start of rehabilitation. Previously, after the surgeon prescribed the need for respiratory rehabilitation, patients had to wait days or weeks until they were assessed by the physiatrist, who gave instructions to the physiotherapists, who finally executed the rehabilitation program. With the introduction of Fissios App©, the situation has changed: the patient can perform respiratory rehabilitation without having to travel to the hospital, and from the first day, the surgeon indicates it.

Most cancer patients prefer to perform exercise programs in a home-based environment [22,23]. Transportation problems and related costs are the biggest barriers to participation in a facility-based intervention [7]. The practice of healthcare supported by information and communication technologies (e-Health) has been increasing in recent years, and thanks to technological advances, we are in a position to expand perioperative exercise programs beyond current limitations [24]. Apps can help people with exercise, diet, and changing their behavior. They may serve as a handy tool to evaluate and motivate smartphone owners who have limited access to healthcare [25]. Fissios App© allows patients to perform physiotherapy exercises completely remotely without time or location limitations and has the possibility of activating daily notifications on the patient’s device to increase adherence to this intervention.

One limitation with the use of apps and smartphones in patients is the need to have a compatible device and that patients know how to use it. To mitigate this limitation, we created an app compatible with iOS and Android devices, available for free download on these stores, and patients received all information for set-up, verbally and written in a document; also, we created a web app version [26]. In our study, sometimes, we needed more time to explain to the patient how to download and use the app. This could be a limitation in other departments, where the medical visit time is very reduced. Providing explanatory documents can improve this situation (Supplementary Material Files S1 and S2) by explaining the whole process to the patient with less time consumption. Another limitation is that the app does not allow the sharing of data stored on devices (offline and noninvasive app). For this reason, a trained physiotherapist verified the use of the app on patients’ devices when they attended face-to-face sessions at the hospital. Although it was a nonrandomized study, we used consecutive nonprobabilistic sampling, and all patients could be part of the intervention group. In addition, we calculated the sample size based on the incidence of PPCs in our department and the expected reduction in the number of events, and we were able to gather two controls for each patient in the intervention group. Finally, we created a binary logistic regression model to adjust for the effect of other confounders and verify the independent influence of the use of Fissios App© on the outcomes. Our work is original and prospective and includes a large number of patients from the same specialty. Fissios App© is a novel tool that could be implemented in routine clinical practice, as it is safe, effective, and useful for patients. For healthcare professionals, it is a tool with scientifically endorsed content, and for healthcare systems, it does not represent any expense. On the contrary, its use could improve postoperative outcomes and may reduce waiting lists in some hospital processes, such as respiratory physiotherapy care.

Our group intends to improve the scientific impact generated by this study, providing Fissios App© with a real-time database, including patient activity monitoring, that could be used in a randomized multicenter clinical trial and avoid possible selection biases that may have occurred in this study. Thus, we will try to validate the association between the uses of this tool and the improvement in postoperative outcomes in thoracic surgery with sufficient scientific rigor. We would also like to test whether the introduction of these new technological tools can replace the attendance of face-to-face sessions or must continue to be used as a complement.

## 5. Conclusions

This study allowed us to draw two important conclusions: The performance of home-based respiratory physiotherapy exercises improved the results of an in-hospital face-to-face respiratory physiotherapy program. And Fissios App© was useful as a complementary tool for a face-to-face respiratory physiotherapy program, improved clinical outcomes after surgery, shortened hospital LOS, and decreased the incidence of PPCs and the probability of developing them.

## Figures and Tables

**Table 1:** Exercises and medical advice included in Fissios App©.

Element	Objective
Exercise	
Basic position (30 s)	Correct position sitting in chair
Nasal and mouth breathing (30 s)	Correct intake and output air during exercises
Abdominal–diaphragmatic ventilation (10 repetitions)	Strengthen the diaphragm, rectus abdominis, abdominal obliques, and transverse abdominis muscle
Lung expansion (10 repetitions)	Strengthen the diaphragm, external intercostals, trapezius, sternocleidomastoid, and scalene muscles
Arm exercises (10 repetitions)	Strengthen the diaphragm external intercostals, trapezius, sternocleidomastoid, and scalene muscles
Sigh and hug (10 repetitions)	Forced expiration and secretions mobilization
Incentive spirometer (10 repetitions)	Encourage long, slow, and deep breaths
Voluntary cough maneuver (3 repetitions)	Forced expiration and secretions mobilization
Effective cough (3 repetitions)	Forced expiration and secretions mobilization
Walking (10 min)	Early mobilization and aerobic activity
Medical advice	
Breathing exercises	Encourage performing of the breathing exercises, establishing a routine, and continuing to do usual physical activity
Stop smoking	Inform patients of the importance and possibility of stopping smoking before surgery
Oral care	Recommendation for adequate oral care and explanation of the relationship with postoperative complications
General points	Information to prepare for surgery, including fasting and usual medication
Physical exercises	Encourage putting into practice the exercises learned before surgery and performing the routine
Out of bed!	Explain the relationship between delaying getting out of bed and postoperative complications
Do you feel pain?	Inform the need to control pain and the importance of relieving it
Balanced and healthy food	Promote a healthy diet and prevention of microaspirations
Immediate postoperative period	Describe the normal postoperative period of a patient during the first 48 h in hospital

**Table 2:** Baseline and surgical characteristics of the patients undergoing surgery.

Variable	Intervention Group (*n* = 131)	Control Group (*n* = 262)	*p*-Value
Male sex (*n*, %)	79 (60.3%)	147 (56.1%)	0.427
Age, y	62 (51–71)	65.5 (55–73)	0.029
Smoking status (*n*, %)			0.947
Unknown	25 (19.1%)	51 (19.5%)	
Nonsmoker	18 (13.7%)	38 (14.5%)	
Former smoker	29 (22.1%)	63 (24%)	
Current smoker	59 (45%)	110 (42%)	
ASA grade (*n*, %)			0.861
I	21 (16%)	34 (13%)	
II	73 (55.7%)	148 (56.5%)	
III	35 (26.7%)	76 (29%)	
IV	2 (1.5%)	4 (1.5%)	
Lung parenchyma resection (*n*, %)	115 (87.8%)	190 (72.5%)	0.001
Surgical approach			0.253
VATS (*n*, %)	67 (51.1%)	118 (45%)	
Conventional (*n*, %)	64 (48.9%)	144 (55%)	

Numeric variables are expressed as medians and interquartile range (25th to 75th percentile). Categorical variables are expressed as numbers and percentages of total: VATS, video-assisted thoracic surgery.

**Table 3:** Comparison of outcomes between patients in both groups.

Variable	Intervention Group (131 Patients)	Control Group (262 Patients)	*p*-Value
LOS (d)	3 (2–5)	4 (3–6)	<0.001
PPCs (*n*, %)	16 (12.2)	63 (24)	0.006
Pneumonia (*n*, %)	7 (5.3%)	27 (10.3%)	0.099
Air leak >5 days (*n*, %)	4 (3.1%)	22 (8.4%)	0.004
Atelectasis (*n*, %)	3 (2.6%)	4 (1.5%)	0.590
30-d mortality (*n*, %)	0 (0)	3 (1.1)	0.219

Numeric variables are expressed as medians and interquartile range (25th to 75th percentile). Categorical variables are expressed as numbers and percentages of total: LOS, length of stay; PPCs; postoperative pulmonary complications.

**Table 4:** Adjusted analysis of patient variables related to PPCs.

Variable	Category	*n*	OR	CI 95%	*p*-Value
Sex	Male	167	2.3	1.3–4.2	0.003
	Female	226	1		
Age	Numeric		0.9	0.9–1.01	0.711
Surgical approach	VATS	185	1	1.4–4.49	<0.001
	Conventional	208	2.5		
Lung parenchyma resection	Yes	305	4.2	1.76–10.21	<0.001
	No	88	1		
Time of use of Fissios App©	No use	262	1	0.17–0.78	0.009
	>4 weeks	77	0.36		

VATS, video-assisted thoracic surgery; PPCs, postoperative pulmonary complications.

## Data Availability

All data were collected in an individual document for each patient. All documentation is stored securely in the office of the Thoracic Surgery Department of the Hospital Clínico San Carlos. Only department members can access this office.

## Supplementary Materials

The following supporting information can be downloaded at: https://www.mdpi.com/article/10.3390/jcm12216774/s1, File S1: Patient information; File S2: Information sheet to download and install Fissios App.
