# Supplementary material for: The Use of Fissios App© as a Complement to a Face-to-Face Respiratory Physiotherapy Program versus an Attendance-Only Face-to-Face Physiotherapy Program in Patients Scheduled for Thoracic Surgical Procedures Reduces the Risk of Developing Postoperative Pulmonary Complications—A Quasi-Experimental Study
Source: J Clin Med. 2023 Oct 26;12(21):6774. doi: 10.3390/jcm12216774 (PMC10650653; doi:10.3390/jcm12216774)
Supplement: Supplementary file 1 [file jcm-12-06774-s001.zip › File S1. Patient information_complementary material.pdf]

PATIENT INFORMATION FOR THE STUDY

INTRODUCTION:

Respiratory physiotherapy as part of a pre-operative physical training program may reduce the risk of developing post-operative complications and improving post-operative results. During the post-operative period, performing physical exercise has also been proved to increase the capacity for exercise, improve health-related quality of life and reduce the feeling of breathlessness. **Fissios App©** is a tool created by by a multi-disciplinary working group composed of thoracic surgeons, physiotherapists and a physiatrist, It contains a structured respiratory physiotherapy program with 10 exercises (including aerobic activity and respiratory muscle training) and 40 multidisciplinary perioperative medical advice

VOLUNTARY PARTICIPATION:

You should know that your taking part in this study is voluntary and you may decide NOT to take part. If you decide to take part, you can change your mind and withdraw your consent at any time. This will not alter your relationship with your doctor or lead to any detriment in terms of your healthcare.

AIM:

The objective is to implement the use of Fissios© as a complement to a face-to-face respiratory physiotherapy program in a group of patients scheduled for a thoracic surgery procedure and determine its efficacy to improve postoperative outcomes in terms of reducing postoperative pulmonary complications and shorten length of hospital stay.

DESCRIPTION OF THE STUDY PROCEDURES:

If you decide to participate, you will receive more detailed instructions on how to download and install Fissios App© (complementary material). You will follow the same protocol stablished in our department: After an evaluation by a physiatrist, who prescribes the exercises, you should attend one-hour, face-to-face respiratory physiotherapy sessions taught by physiotherapists at hospital, five times a week, before the planned surgery and continued with the same frequency during the postoperative period until discharge. The physiotherapy sessions included breathing exercises, usage of incentive spirometry, and coughing exercise. The control group will only attend face-to-face respiratory physiotherapy sessions, the intervention group will attend to these sessions and, also they will be encouraged to use Fissios App© before

surgery, accomplish the medical advice and perform the respiratory physiotherapy program included.

EXPECTED BENEFITS:

The performing of respiratory physiotherapy exercises can reduce the number of peri-operative complications and improving of post-operative results. Fissios App was designed to help you perform these respiratory exercises and give a few peri-operative medical recommendations. Using Fissios App can improve the performing of respiratory physiotherapy exercises. It may occur that using Fissios App does not have any added benefit to the performing of a face-to-face respiratory physiotherapy program.

POSSIBLE ALTERNATIVE TREATMENTS:

Respiratory physiotherapy exercises can be performed individually. However, an evaluation by health professionals and an individualized prescription of the exercises is recommended. A program of respiratory physiotherapy exercises that deals with activities such as: aerobic exercises, breathing exercises and training inspiratory muscles can be performed face-to-face supervised by health professionals with or without use of the Fissios App.

CONFIDENTIALITY:

All the data in regard to you and your health covered over the course of the study will only be used to perform this; in the event of other possible future studies related to your pathology, these will be approved in advance by a Research Ethics Committee. Your data will be managed under the strictest confidentiality: your name and your medical information will be replaced by a code so that no individual participant can be identified. The only person who will have access to the key code is the study's principal investigator. You also have the right to limit the processing of data that are incorrect, request a copy or that the data you provided for the study, to the extent they are applicable, be sent to a third party (portability). To exercise these rights, contact the study's principal investigator.

NOTE: This document contains confidential patient information, whereby it should be stored by the investigator, together with the remaining study documentation. If the patient so wishes he may receive a copy of this same document.
