# Supplementary material for: The Use of Fissios App© as a Complement to a Face-to-Face Respiratory Physiotherapy Program versus an Attendance-Only Face-to-Face Physiotherapy Program in Patients Scheduled for Thoracic Surgical Procedures Reduces the Risk of Developing Postoperative Pulmonary Complications—A Quasi-Experimental Study
Source: J Clin Med. 2023 Oct 26;12(21):6774. doi: 10.3390/jcm12216774 (PMC10650653; doi:10.3390/jcm12216774)
Supplement: Supplementary file 1 [file jcm-12-06774-s001.zip › File S2. Information sheet to download and install Fissios App_Complementary material.pdf]

INFORMATION SHEET TO DOWNLOAD AND INSTALL FISSIOS APP

This document is informative.

Disponible en el App Store

<u>Instructions to download and install Fissios. Apple® devices.</u>

(Requires iOS 11.0 or subsequent. Compatible with iPhone, iPad and iPod touch).

The way to download **Fissios** is to enter the **App Store®**. The **App Store®** is the **Apple®** applications shop. Inside you can look for and download **Fissios**. There are only three very simple steps.

1. Look for the **App Store®** icon on your device's screen and enter the store.
2. The **App Store®** opens in the lower part, click on the icon **Search**. Type in **Fissios** and the search will begin.
3. Click on the button **install** and **Fissios** will start to download.

You can also scan the QR code:

DISPONIBLE EN Google play

<u>Instructions to download and install Fissios. Android® devices.</u>

(Requires Android versions 4.4 and above).

The way to download **Fissios** is to enter the **Play Store®**. The **Play Store®** is the **Android®** applications shop. Inside you can look for and download **Fissios**. There are only three very simple steps.

1. Look for the **App Store®** icon on your device's screen and enter the store.
2. The **Play Store®** opens at the top of the page. Type in **Fissios** and the search will begin.
3. Click on the button **install** and **Fissios** will start to download.

You can also scan the QR code

To extend the information, consult the website and social networks.

www.fissiosapp.com/e-mail: fissiostoracica@gmail.com

Twitter: @fissiosapp

Facebook: Fissios App

Google +: Fissios App

INFORMATION SHEET TO CONFIGURE AND USE FISSIOS 3.0

Dear Patient, this sheet gives some instructions on the correct configuration and initial steps when using Fissios. It is an App that is simple to use and interactive. Feel free to read all the sections we have created and do not forget to perform your respiratory exercises.

Personalize Fissios

You need to insert a name or nickname and the likely date of your surgery. Fissios will tell you how many days to go for your surgery and will send you personalized notifications with important information. You can modify your name and date of your surgery at any time (*When modifying your name the time counter will restart, do not change this if this is unnecessary*).

Note: In accordance with that set out in the EU General Data Protection Regulation 2016/679 of the European Parliament and Council, we hereby notify you that the option to personalize Fissios is free and not associated with any user's personal data. By agreeing to take part in this study, no personal data will be processed. Only anonymous data in regard to use and interaction with Fissios will be processed. This application does not necessarily require access to internet and does not access personal data from your mobile device. There is no risk of sending personal information to third parties via the application.

Initial screen

You will find a days counter and Fissios tells you how many days are left before your surgery. You will also find all the windows that comprise the app (pre-operative advice, post-operative advice, respiratory physiotherapy exercises, warnings, modify my data). You can choose any option.

Pre-operative advice, post-operative advice, warnings

These sections are comprised of information as advice. Choose the option you wish to consult and a window drops down with information that would be useful to prepare you for surgery and the post-operative period. Put into practice all the advice that have been compiled in the app.

Respiratory physiotherapy exercises

You can choose between performing the entire routine of exercises or performing an exercise separately. The exercises screen is comprised of an animated image that shows the correct way to perform the exercise. The lower part of the screen gives a step by step outline of the correct way to perform the exercises. Use the buttons aimed at this screen. The start button sets in motion the clock that counts the seconds or repetitions that comprise the exercise. *(You need to click the "start" or "play" button (▶) so that Fissios records and counts the time used to perform exercises).* The restart button enables restarting the clock to repeat the exercises.
